# Dr. Nicholl on Disordered States of the Cerebral Structures, in Infants

**Published:** 1821-09-01

**Authors:** 


*Practical Remarks on Disordered States of the Cerebral Structures, occurring in Infants.* By Whitlock Nicholl, M.D. M.R.I.A. F.L.S. &c. &c. One vol. small octavo, pp. 94. London, 1821.

Dr. Nicholl is an acute observer and a close reasoner—and although we think his distinctions and descriptions are too minute for general application to clinical practice by the great body of the profession, yet we believe that his observations will be acceptable to those of the higher orders of the faculty who have zeal and ablility to examine and apply them.

Dr. Nicholl is perfectly correct when he observes, that consequences of a diseased state are often confounded with the causes from which they arise—a remark which is particularly applicable to affections of the cerebral structures—more especially hydrencephalus. We believe him correct in considering the effusion, in a majority of cases, as the sequela or effect of previous disease.

"There is a state or condition of the cranial brain in infants, which may be called *a state of irritation*, an *irritated state*, or, in one word, *erethism*. What this peculiar condition of the cerebral structure is, I cannot explain. It is a state, distinct from that which is called Inflammation of that structure, for it may exist without any perceptible increase of the quantity of blood that flows through the cerebral blood-vessels. It is a state, under which, inordinate effects arise from ordinary impressions upon different parts of the nervous system. In its perfect form, and under a high degree of it, it is a highly sensitive condition of the cranial brain, a condition the very reverse of that under which sleep occurs. Under such a condition of the cranial brain, the child is wakeful, scarcely ever sleeping; it is attentive to every sound, and to every object of sight; its temper is irritable; the retina is highly sensible to light, so that the child quickly winks if its face be turned towards the window, or towards a candle; the pupil is, in many instances, more or less, contracted, but this is not always the case; the limbs are much in action; the head is often moved about, or is shaken from side to side; the child cries without any apparent cause, and it is soothed only by tossing it, by carrying it about, by putting it to the breast, or by letting it suck the cheek of the nurse, or its own fingers; the secretion of tears is, in many instances, increased, causing suffusion of the eyes, and redness of the edges of the tarsi; the secretion of the schneiderian membrane may be increased, causing a stuffed state of the nasal passages, producing sneezing, and exhibiting the appearance of that state which is, popularly, called *a cold*; the bowels are, in many cases, relaxed, yet no disordered state of the stools may appear. During such a state as I have described, there may be a degree of animation, and a quickness of observation, much beyond what are commonly met with in children of the same age. So that, although a morbid condition of the cranial brain be present, the child may be considered as particularly healthy, on account of its being wakeful and lively, and sensible to the most trifling impression. But it frequently happens, that an attentive observer may detect other symptoms; the child may start in its sleep; it may be very readily awakened; when awake, it may start at the slightest noise, as at the shutting of a door, at the moving a chair, at passing the finger over the wicker-work of its cradle, or on being slightly moved, or if touched gently; a sudden frown may pass over the forehead and may quickly disappear; the eyes may be closed irregularly, or alternately, or a winking of one eye, or frequent winking of both eyes, or a firm closing of both eyes, may be, from time to time, detected; the hand may be raised frequently to the head; the child may cry, without any evident cause, as if it were pricked with a pin; at other times, it may shriek; the fists may be clenched, the thumb being bent in, and laid flat across the palm of the hand, the fore-arms being bent upwards on the arms. Should a similar condition of the spinal brain be present, the child may be bent backwards, presenting a state of *opisthotonos;* its legs may be drawn up, while the head is thrown backwards. 13.

Such a state, Dr. N. thinks, constitutes what may be termed *sensitive erethism.* An increase of heat in the head or body may sometimes, but by no means constantly, attend this cerebral erethism.

There is another form of infantile erethism characterized by want of animation, fretfulness when roused, want of sleep, and yet "a state that can hardly be called waking," indifference to surrounding objects, pallor and chilliness of the body, rolling of the eyes, plaintive moaning or shrieking, jactitation of the hands and other minor symptoms, which our author denominates *torpid erethism.*

*Scrofulous* children have generally the greatest tendency to cerebral erethism, and where this tendency exists, the slightest irritation of the nervous system will call it into action.

The etiology of this state is extensive; but among the most frequent causes are impressions on the extremities of the cerebral nerves. Hence dentition, worms, depraved visceral secretions, drastic purgatives, inactive liver, congestion of the hepatic system—"in short, whatever causes great irritation in any part of the body, whether in any of the great cavities, or on the surface of the body, may produce an erethismal condition of that brain with which the nerves of the irritated part principally communicate."

In the milder forms and earlier stages of this affection, the symptoms which it produces may be great wakefulness, great sensibility to slight impressions, with restlessness and high animation—symptoms which are generally hailed by nurses as proofs of health and vigour of the child, and consequently the disease is often overlooked. It is also often overlooked when it happens to arise during the presence of disorder of some other part, or where its assemblage of symptoms veils the true seat of the disease.

"Thus, such a condition of cerebral substance, in what way soever it arises, may produce an assemblage of symptoms to which the general term *fever* is applicable; or it may cause convulsive affections, or sundry other derangements of functions; yet our attention may be engrossed by these several effects, while the cause from which they arise is entirely overlooked." 22.

The spinal brain (as it is termed by our author) is also liable to the erethism in question, which is sometimes propagated from one brain to the other, or affects them alternately. When it exists in the spinal brain alone, the principal symptoms to which it gives rise, according to Dr. Nicholl's observations, are :—

"Restlessness; irregular and incessant movements and twitchings of the extremities; writhing of the body; hurried respiration; and irregular action of the alimentary canal." 23.

This state may be induced by injuries, vicissitudes of temperature, by impressions on the nerves of the abdominal viscera, "and especially by morbid states of the alimentary canal"—in short, from irritation of any of those nerves communicating freely with the spinal brain.

Erethismal states of the cerebral structures are not confined to infancy. They present themselves very frequently in adults, giving rise to symptoms closely resembling those observed in children.

"Irritability of temper, inability to bear the effects of the most trifling sounds, wakefulness, restlessness, febrile symptoms, the whole train of what are called *nervous symptoms*, convulsive affections; these may, severally, be traced, in many adults, to erethismal conditions of one, or both, of the cerebral structures." 27.

As an increased circulation in the cerebral vessels tends to the production of these erethismal states, so does erethism, on the other hand, tend to increase the circulation. The two states united, our author thinks, constitutes what is termed INFLAMMATION—a state including erethism, of course; but erethism of the cerebral structures may exist independently of any perceptible increase of the quantity of blood in circulation. High erethism and circulation in infants, constitutes, Dr. N. imagines, acute inflammation, and less sensitive erethism, with moderate circulation, subacute inflammation. A moderate increase of circulation, with torpid form of erethism, may possibly constitute chronic inflammation.

"Inflammation of the cranial brain in infants is characterized by the following symptoms: redness of the conjunctiva; highly-contracted pupil; great intolerance of light, and of sounds; suffusion of the eyes; great restlessness; wakefulness; charged state of the blood-vessels of the head; throbbing of the arteries of the neck and head; dry, hot mouth; thirst; startlings; shriekings; hurried, unmeaning manner, and expression, of countenance; pulse, throbbing, full, strong, increased in frequency." 29.

The spinal brain may also be the seat of the phlogosis; but simple *plethora* of the cerebral vessels our author characterizes by the following symptoms:—

"Increased heat of the head; a full eye; a redness of the countenance, with a want of animation; a heavy, listless, state;* indisposition to move the head; uneasy sense of fulness in the head, causing the child to seek support for the head; giddiness; shaking of the head; uneasy respiration; sighing; the pupil either of a natural size, or rather dilated; sickness; loss of appetite; heat, and dryness of the mouth and skin; an inactive state of the bowels; a sensibility to impressions, but a heedlessness of them; a turgid state of the vessels of the head; the pulse not much accelerated, full, perhaps oppressed." 31.

Dr. Nicholl distinguishes a plethoric state of the veins of the head by the term *congestion;* in which state, he observes, there is general coldness of the child's body—jactitation of the head—comatose disposition—prominence and fixedness of the eyes—dilatation of the pupils—grinding of the teeth—irregular action of the facial muscles—indistinctness of vision—insensibility, more or less complete, to impressions—strabismus—torpor of bowels—scanty or suppressed secretion of urine—slow oppressed pulse—slow laborious respiration.

Irritation of the cranial nerves, as of the nerves of other structures, disposes the exhalents of the part to throw out an increased flow of fluid. When the irritation, however, is on the anti-cerebral extremities of the nerves, for instance, in the liver or other abdominal organs, the effusion may be so gradual in the cavities of the brain, as to take place to a considerable extent, without marked or decided symptoms.

We cannot follow our author through his almost endless distinctions and descriptions, and our reasons for not following him, he himself shall furnish.

"I think that I am correct in stating, that each of these conditions of the cranial brain may exist as a distinct affection, and that, in the pure, uncombined, form of each of these states, each condition has distinguishing characters, by which its presence is denoted. But, when we review these various conditions, and consider how they may be blended; when we look over the list of causes from which they may severally arise, and see how many of the causes of each condition may exist at the same time; and when we take into the

* Sic summè plethoricos sæpe invenimus debilissimos; post largam vero sanguinis missionem redeunt illicò pristinæ corpori vires. *Van Swieten, Comm.* II. 270.

account, the influence which various modes of treatment have upon disease, how much they alter its features, and modify and interrupt the train of symptoms; we shall be prepared to meet with appearances very different from those which are *traced upon paper*, and we shall not be surprized at being called upon to treat cases, in which there is an assemblage of symptoms, that baffle all our attempts at classification, and defy all nosological distinctions." 56.

We are no advocates for rendering medicine so simple a science, that he who runs may read, and he who reads may easily comprehend; but we do think that an opposite extreme may be run into, and that hair-breadth differences and distinctions may be so multiplied that the *actual clinical practitioner* will be frightened from all attempts to gain information from the writings of others.

In the therapeutical portion of this little volume we found much to commend—and recommend to our junior brethren.

The most common affection of the cranial brain in infants is *erethism*—the other conditions of that organ being, in many instances, consequences of such erethism. Where there is predisposition in the child, marked by precosity of intellect, and the physical characters of scrofula—and especially where others of the family have laboured under cerebral affections, we must cautiously guard against those exciting causes of cerebral erethism, which are well known to all intelligent practitioners. The diet in particular should be attended to. Our author justly observes, that "nurses never take the digestive powers of a child into consideration, nor do they ever seem aware that the process of digestion is necessary to convert food into aliment." When an infant is brought up by hand, it is difficult to restrain the nurse from exhibiting food of too rich quality. The stomach and duodenum become overloaded—the chyme becomes unhealthy—the liver congested—and sympathetic irritation propagated to the brain. On the other hand, it ought not to be forgotton that—

"The state of the alimentary canal in infants is much influenced by the condition of the cerebral structures. Thus, an altered state of the cranial brain may induce sickness, disordered secretion of the fluids which flow into the alimentary canal, and a relaxed, or constipated, state of the intestines. Disorder of the spinal brain may also quicken or retard the peristaltic action of the bowels." 67.

On the hepatic affections, so very common in children, Dr. N. makes many judicious remarks, but not particularly novel.

"Calomel," says he, "is given to promote the secretion of bile; it is also given where that fluid is too abundantly secreted. The mode of exhibiting it must be adapted to the particular state; in the former case, small doses of it should be given in repeated succession so as to produce the specific effects of mercury on the liver; whereas, if we want to remove from the alimentary canal an inordinate quantity of bile which has flowed into it, we should give one powerful dose, so as to produce its effects as a strong purgative." 73.

In scrofulous constitutions we should be careful, of course, in the administration of this remedy.

"In such children, if we want to promote a more copious secretion of bile, it will be more prudent to endeavour to attain that object by employing other remedies, such as nitro-muriatic acid, externally and internally, small doses of sulphat of potash with decoction of aloes, wine of aloes, or extract of taraxacum. Of if we find it necessary to employ mercury to promote the action of the liver, the mercurial pill, the grey oxide of mercury, and the hydrargyrus cum creta, are less objectionable forms of that mineral. These several means may be assisted by the warm salt bath, by friction with salt, by applying a blister over the region of the liver, and by clothing the child in flannel." 74.

Dr. N. repeats the observation of some practitioners that the stools resembling "*stewed spinach or boiled laver*," are produced by the calomel prescribed. All we can say is, that *facts* are adverse to the *theory*. We have repeatedly seen these stools where no calomel had been given, and we have seen them dislodged by scammony, jalap, and other purgatives than mercurial. But it is curious to observe how an opinion taken up by an individual, will pass current, without examination, through a whole generation of writers, and after all prove entirely without foundation. How is it, we ask, that calomel taken when a man or child is in health, will not produce *spinach* in the intestines?

To obviate the cerebral erethism, the child should be frequently carried into the open air—"its *body* and head should be frequently sponged with cold water, and it should be immersed night and morning in *cold* or tepid water." We much question the propriety of cold water to the surface of the body when an internal organ of such vital importance as the brain is in a state of erethism. We would not prescribe such a measure ourselves; but let others do as they please.

"If the wakefulness still continues, small doses of pulv. ipecac. comp. may be given from time to time."* 78.

---

"* The dose of the opiate must be regulated by its effect. In treating erethismal states of the cerebral structures with opiates, we must

The pulv. ipecac. comp. may be advantageously combined with James's powder—especially in that form of the disease denominated *sub-acute* inflammation. Cold evaporating lotions to the head—cool, quiet, darkened room—elevation of the head and shoulders—diuretics of nitre, with acetate of potash and nitric ether—aperients—tepid bath—leeches to the temples, where the child is robust—lancing the gums—constitute the principal remedial measures of our author. When the disease is evidently accompanied with plethora, or, in other words, *inflammation* of the brain, local bleeding from the head, or even general blood-letting, must be carried to a greater extent than in simple erethism. Besides the means already pointed out—

"The bowels must be purged by calomel and saline purgatives, after which, James's powder combined with calomel and nitre may be given in repeated doses. The child must be kept in a quiet, darkened room, free from noise and disturbance.

"It may happen, when an inflammatory state of the cranial brain has existed, that the remedies made use of may remove the plethoric state of the cerebral blood-vessels, while an erethismal state of the brain yet remains. In such cases, it will be proper to give the pulvis ipecac. comp. in repeated doses, combined with James's powder and nitre, to which small doses of calomel, or of the hydrarg. cum creta, may be added, and the other rules laid down for the treatment of erethism of the cranial brain must be strictly attended to, as long as that state continues; for if the erethismal state of that organ be not allayed, the child will continue wakeful, restless, and irritable; and we may expect, that the plethoric state of the cerebral blood-vessels will, sooner or later, return; and that the child will, at length, be worn out, or permanent mischief may be established. And if, after all the marked appearances which indicate the existence of an erethismal state, have vanished, the child is wakeful, or very irritable, we must still procure rest, and allay irritation, by repeated doses of the Dover's powder, in such quantities, and at such times, as may be requisite; recollecting, that there still exists a tendency to the revival of an erethismal condition of the cranial brain, which circumstances, apparently trifling, such as the long-continued absence of sleep; any error of diet; an unhealthy condition of the alimentary canal, or in the liver; or any irritation;—may suddenly call into action," 85.

---

recollect that opium produces opposite effects in different cases, and in different doses. Opium may allay irritation of those structures, it may produce complete torpor of them, or it may act upon them as a stimulant. Our object in exhibiting opium in erethismal states must be, to allay irritation, we must therefore so administer it as to produce this effect, and this end will be best attained by giving the pulv. ipecac. comp. in small repeated doses. This is a point too often overlooked."

We have much overstepped the boundaries within which we meant to confine the analysis of this little volume. If we have differed with the author on some few points, and if we have thought him disposed to too much refinement, both in symptomatology and pathology, we have not ceased to admire his zeal, and respect his talents. We have no doubt but that Dr. Nicholl will contribute much to the advancement of medical science, and stand conspicuous among its present cultivators.

